# Complete genome sequence of *Bacillus subtilis* strain MARUCo01 isolated from marine sediments of the Indian Ocean in Bagamoyo, Tanzania

**DOI:** 10.1128/MRA.00473-23

**Published:** 2023-08-22

**Authors:** Reuben Maghembe, Edward Moto, Abdalah Makaranga, France Mdoe, James Mpemba, Maximilian A. K. Magulye, Deogratius Mark, Andrew Mtewa

**Affiliations:** 1 Biological and Marine Sciences Unit, Faculty of Natural and Applied Sciences, Marian University College, Bagamoyo, Tanzania; 2 Department of Biological Sciences, Faculty of Science, University of Botswana, Gaborone, Botswana; 3 Department of Microbiology and Immunology, Faculty of Biomedical Sciences, Kampala International University Western Campus, Kampala, Uganda; 4 Department of Biology, College of Natural and Mathematics Sciences, University of Dodoma, Dodoma, Tanzania; 5 Department of Biochemistry and Physiology, St. Francis University College of Health and Allied Sciences, Ifakara, Tanzania; 6 Department of Immunology and Molecular Biology, School of Biomedical Sciences, Makerere University, Kampala, Uganda; 7 Department of Disease Control, Tanzania Agricultural Research Institute (TARI), Dar es Salaam, Tanzania; 8 Chemistry Section, Department of Applied Sciences, Malawi Institute of Technology, Malawi University of Science and Technology, Limbe, Malawi; University of Delaware College of Engineering, Newark, Delaware, USA

**Keywords:** genomics, *Bacillus subtilis*, strain MARUCo01, genome analysis, average nucleotide identity (ANI)

## Abstract

*Bacillus subtilis* has emerged as a species with potential for versatile nonribosomal peptides and polyketides of therapeutic importance, including antibiotics. From our molecular bioprospecting project, we report a full genome of *Bacillus subtilis* strain MARUCo01 locally isolated from sediments of the Indian Ocean along the coast of Bagamoyo in Tanzania.

## ANNOUNCEMENT

*Bacillus subtilis* is species of widely distributed bacteria associated with multiple benefits to human, including the production of antimicrobials and the potential for probiotics (1). These, among other benefits, are a trigger for increased search for and manipulation of novel *Bacillus subtilis* strains in the expression of various secondary metabolites as drug scaffolds. In our ongoing bioprospecting project, as described in our recently published work (2), surface sediment samples were collected from a mangrove forest area in the Indian Ocean shore along the Bagamoyo Coast in Tanzania (6°25*'*32.5*"*S 38°54*'*08.1*"*E, https://www.google.com/maps/search/?api=1&query=-6.4256828,38.9022509). The sediment samples were collected in sterile plastic bottles and kept there at 4°C. A piece of the sediment (about 5 g) was suspended in 200 mL of 0.80% NaCl, followed by serial dilutions (10^−1^ to 10^−6^) with phosphate-buffered saline (PBS) (pH 7.2), and the subsamples of these dilutions were plated on nutrient agar (NA). The individual colonies were then picked and restreaked on NA and incubated at 28°C for 48 h before DNA extraction.

Cells were picked directly from the plated colonies, resuspended in PBS, and the genomic DNA was extracted using a ZymoBIOMICS DNA cvMiniprep Kit (ZR D4300) based on the manufacturer’s guide. Then the TruSeq DNA PCR-Free Kit and TruSeq Nano DNA Kit were used to build libraries, and the Ilumina Novaseq 6000 platform (2.5 G bp) was used to sequence the entire genome, producing small paired-end reads with an average length of 151 bp. Raw reads were quality controlled using FastQC and Trimmomatic (v 0.38) under the following criteria: minimal sequence length for both reads prior to a sequence pair removal is 20 bp, and the minimal adapter overlap (stringency) is 1 bp. With the use of the Unicyler pipeline (v. 0.4.8) (3), the reads were *de novo* assembled into contigs, which were then checked for species contamination with the ContEst16S pipeline (4). A chromosome was then generated using CONTIGuator (v.2.7.40 (5) and then annotated using the best-placed reference protein approach (set; GeneMarkS-2+) of the Prokaryotic Genome Annotation Pipeline (PGAP v.6.3; https://www.ncbi.nlm.nih.gov/genome/annotation_prok/). The taxonomic placement was predicted using the Microbial Genomes Atlas (MiGA) (6) and then confirmed with average nucleotide identity (ANI) calculation with FastANI (7).

From whole-genome Illumina sequencing, 19,041,058 reads were generated, which, upon quality control, were reduced to 12,490,734. *De novo* assembly resulted in 29 contigs (a total of 4,058,030 bp) with N50 of 995,325 and GC content of 43.7%. PGAP annotation revealed 4,206 genes, with 4,037 coding sequences. Combing MiGA taxonomic approach with FastANI estimation, the MARUCo01 strain was unambiguously placed into the species *Bacillus subtilis*, and the closest relative identified was *Bacillus subtilis* subsp. subtilis str. 168 (NCBI accession number AL009126.3) with an ANI value of 98.74%.

## Data Availability

The genome sequence was submitted to GenBank and can be accessed via the accession numbers CP107539.1 (chromosome), CP107540.1 (plasmid), OP586436.1 (16S rRNA), and BioSample and BioProject accession numbers SAMN31159204 and PRJNA887360, respectively. The assembly and raw read sequence data sets can be accessed through GCA_025837035 and SRS15977960, respectively.
